# Extracellular matrix-induced signaling pathways in mesenchymal stem/stromal cells

**DOI:** 10.1186/s12964-023-01252-8

**Published:** 2023-09-19

**Authors:** Ekaterina Sergeevna Novoseletskaya, Pavel Vladimirovich Evdokimov, Anastasia Yurievna Efimenko

**Affiliations:** 1 Faculty of Biology, Dayun New Town, Shenzhen MSU-BIT University, 1 International University Park Road, Dayun New Town, Longgang District, Shenzhen, Guangdong Province P. R. China; 2 Institute for Regenerative Medicine, Medical Research and Education Center, Lomonosov Moscow State University, Lomonosov Ave., 27/10, 119991 Moscow, Russia; 3 Materials Science Department, Lomonosov Moscow State University, Leninskie Gory, 1, Building 73, 119991 Moscow, Russia; 4 Chemistry Department, Lomonosov Moscow State University, GSP-1, Leninskiye Gory, 1-3, Moscow, Russia; 5 Faculty of Medicine, Lomonosov Moscow State University, Lomonosov Ave., 27/1, 119991 Moscow, Russia

**Keywords:** Mesenchymal stem/stromal cells, Signaling pathways, Extracellular matrix, System biology

## Abstract

The extracellular matrix (ECM) is a crucial component of the stem cell microenvironment, or stem-cell niches, and contributes to the regulation of cell behavior and fate. Accumulating evidence indicates that different types of stem cells possess a large variety of molecules responsible for interactions with the ECM, mediating specific epigenetic rearrangements and corresponding changes in transcriptome profile. Signals from the ECM are crucial at all stages of ontogenesis, including embryonic and postnatal development, as well as tissue renewal and repair. The ECM could regulate stem cell transition from a quiescent state to readiness to perceive the signals of differentiation induction (competence) and the transition between different stages of differentiation (commitment). Currently, to unveil the complex networks of cellular signaling from the ECM, multiple approaches including screening methods, the analysis of the cell matrixome, and the creation of predictive networks of protein–protein interactions based on experimental data are used. In this review, we consider the existing evidence regarded the contribution of ECM-induced intracellular signaling pathways into the regulation of stem cell differentiation focusing on mesenchymal stem/stromal cells (MSCs) as well-studied type of postnatal stem cells totally depended on signals from ECM. Furthermore, we propose a system biology-based approach for the prediction of ECM-mediated signal transduction pathways in target cells.

Video Abstract

**Supplementary Information:**

The online version contains supplementary material available at 10.1186/s12964-023-01252-8.

## Introduction

Tissue structure homeostasis, function, and renewal depend on cellular composition. Generally, terminally differentiated cells define the structure and normal function of tissue and organs, while the adult stem and progenitor cells determine renewal and regeneration potential [1–3]. Stem cell stability is based on the sustainable work of cell signaling pathways, which is controlled by intracellular (non-coding RNAs and transcription factors) and extracellular (growth factors, morphogens, environmental cues) factors [4, 5]. Changes to microenvironment conditions cause the transition of stem cells from a quiescent to an activated state, which initiates proliferation and differentiation [6, 7]. The combination of external microenvironmental factors that support the functioning of stem cells has been labelled a “stem cell niche” [8]. An integral component of the niche is the extracellular matrix (ECM), which provides most of the clues from the microenvironment, i.e., physical characteristics, the conduction of specific signals from structural components, and the anchorage of components to the ECM, e.g., soluble factors (growth factors and morphogens) and matrix-bound vesicles [9]. Thus, the ECM stimulates various intracellular signaling cascades required to maintain the homeostasis of stem cell niches [10]. Herefore, interpreting the research results obtained from cells isolated from their microenvironmental context is complex.

Stem cell niches contain cells that regulate the maintenance of stem cells homeostasis and fate through the secretion of various niche components. Almost in all tissues and organs mesenchymal stem/stromal cells (MSC) play this role being the critical regulators of stem cell niche functioning [11–15]. MSC can secrete a variety of niche ECM components, paracrine factors, and bioactive molecules within extracellular vesicles in response to changes in the microenvironment (e.g., injury). In addition, under activating stimuli, multipotent stem cell subpopulation of MSC is capable of self-renewal of their own pool as well as differentiation, leading to modification of the microenvironment by replenishing deficient components or recruiting other supporting cells to the niche [16, 17]. According to one of the minimal criteria to define multipotent MSC are capable of adipogenic, chondrogenic and osteogenic differentiation in vitro [17]. Until recently, scientists used the term "mesenchymal stem cells" to discuss these cells, but it was considered incorrect due to the collected data proving that the main physiological function of MSC is not exclusively the presence of stem cells [18]. Therefore, the current recommended name is “mesenchymal stromal cells”, and the presence of multipotent stem cells in MSCs should be carefully evaluated using appropriate tests [17]. Nonetheless, all MSC are heavily reliant on the ECM clues, so we focus on these cells to analyze the diversity of ECM-induced signal pathways in postnatal stem cells.

The interaction of cells with the microenvironment, in particular with the components of the ECM, is mediated by specific receptors, leading to the activation of various signaling cascades within the cell and, as a consequence, to changes in its behavior. Looking at the identification of the main receptors and participants in ECM signaling cascades from a historical point of view can help to summarize the knowledge on this issue in the field of matrix biology (Table 1). Even though many receptors and key participants of their signaling cascades have been known for a long time, there are many gaps among the other participants of these cascades, as well as in the case of changing microenvironment and the nature of their network interactions, and for a specific type of cell. This tendency highlights the importance of the analytical reviews covering the mechanisms of ECM-mediated regulation of cell function, here in particular the stem cell differentiation. In parallel with investigation of the ECM, the signaling cascades of postnatal stem cells remain an important issue for cell and matrix biology and regenerative medicine. In this review, we focus on analyzing the data obtained from MSC in vitro and in vivo for a better understanding of the regeneration processes relevant to native postnatal stem cells. Accumulating ECM signaling observations, we suggest a system biology-based approach for examining the predicted networks of such signal transduction pathways on the example of DDR1-initiated signaling in MSC.

**Table 1:** General discovery dates for ECM signaling molecules

Time of discovery	Discovered phenomena	References
1942	The discovery of glycosphingolipids was by the German scientist Ernst Klenk after their isolation from brain tissue	[19]
1980	CD44 was first described as a surface molecule of lymphocytes, platelets, and granulocytes. Currently, it is a receptor primarily for hyaluronic acid, but also for lipoproteins and proteoglycans of the ECM, growth factors, cytokines, and matrix metalloproteinases (MMP)	[20, 21]
1983, 1989	Syndecans were first identified in 1983; a few years later they were named using the Greek term *syndein*, meaning “to bind together”, which emphasized their importance for cellular adhesion to the ECM	[22, 23]
1985, 1993, 1997	The receptor discoidin-1, a lectin, involved in the adhesion, aggregation, and tightly regulated migration of cellular slime mold (*Dictyostelium discoideum*) was discovered. Subsequently, receptor tyrosine kinases with a domain to discoidin homologous were discovered. Investigation of these proteins resulted in the discovery of discoidin domain receptor tyrosine kinase 1 and 2 (DDR1, DDR2)	[24–27]
1985	Transient receptor potential (TRP) channels were found first in *Drosophila* as rhodopsin-triggered phospholipase C (PLC). Later, TRP channels were found in fungi and animals, where they act as mechanosensitive ion channels	[28–30]
1986	Integrins, major receptors that interact with the ECM, were discovered. They were named to represent their participation in the transmembrane glycoprotein complex, that provides conjunction between ECM and the cell actin cytoskeleton	[31]
1989, 1990, 1992	Paxillin, a phosphotyrosine-containing protein, was identified. Furthermore, its role in cell adhesion to the ECM proteins was studied in detail	[32–34]
1996, 2009	Integrin-linked kinase (ILK) was observed as a protein that associates with cytoplasmic tail integrin β1 subunits. However, it was later determined that ILK has a pseudoactive kinase domain and forms the center of protein–protein interactions	[35, 36]
1999	Since its discovery, talin has been considered the only mediator of integrin activation	[37]
2000, 2004, 2009	Subsequently, it was found to be essential for integrin activation and signal transduction of kindlins	[38–40]
2010	Other mechanosensitive ion channels, e.g., Piezo, was described in 2010. Their activation under mechanical forces was reported for several cell types	[41, 42]

## ECM receptor signaling pathways during MSC differentiation

Many ECM components were found in stem-cell niches, including collagens, laminins, fibronectin, and proteoglycans [43], and also paracrine factors affecting the interaction of stem cells with the ECM [44]. The ECM supports the appropriate position of cells within their microenvironment and regulates such properties as proliferation, polarization, migration, and differentiation [45]. Several studies have demonstrated that the ECM directs the differentiation of stem cells into specialized cells of the organ from which it was isolated. This data confirms that the ECM has tissue specificity for maintaining a certain niche [46].

It is presumed that the tissue specificity of the ECM is provided due to differences in the cellular composition of tissue types. Nevertheless, it is known that cells with similar phenotypes and functions isolated from different tissue types differ in the expression profile of ECM proteins. Thus, a comparative proteomic analysis of the ECM, secreted by MSC from bone marrow or adipose tissue, showed the presence of unique sets of proteins produced by each cell type. This allows us to assume that ECM tissue specificity is established at histo- and organogenesis stages and subsequently maintained throughout life [47]. The composition of ECM components, which are distinguished not only among different tissues and organs but also within niches, was confirmed for niches of the intestinal crypt, hematopoietic niche, and limb [48, 49]. Such diversity among ECM components within the niche provides a further indication for stem cell outcomes. Each component supports important functions, from keeping the stem cell in the quiescent state to asymmetric division, migration along to ECM components or soluble factors, and the termination of differentiation [50–52]. These functions are mediated by the activation of a specific signal cascade. The interaction of participants in the cell signaling pathways, including MSC, with the microenvironment is carried out using special molecules, the most important of them are discussed below.

### Integrins

The main class of ECM receptors is integrins, heterodimeric proteins comprising α and β subunits. There are 18 determined α-subunits and 8 β-subunits in humans, and these are responsible for recognizing ECM proteins and their physical properties (e.g., stiffness and stretching), and for intercellular communications. Some reviews can provide more detailed information on modern representations of the structure of integrins [53–55].

Integrins realize bi-directional signaling. The high-affinity interaction between integrins and their ECM ligands activates the “outside-in” signaling pathway. Then, focal adhesion kinase (FAK) and Src-mediated phosphorylation of the integrin adhesion complex (IAC) and cytoskeletal components initiate intracellular molecular reorganization and phosphorylation events among many adapters [56]. Crosstalk between FAK and Src kinases provides signaling pathways induced by mechanical forces and RTK signaling, leading to control of stem cell fate transitions [57]. For example, initiation of FAK/Src/Rac1-mediated myosin IIA recruitment into FAs increased the osteogenic commitment of human bone marrow MSC [58]. On other occasions, intracellular signals interact with the cytoplasmic tails of integrins, which leads to conformational changes in the extracellular ligand binding domain. This mechanism fine-tunes the control of ligand affinity [56, 59].

Integrins are considered crucial receptors for stem cell functioning. Various integrins are widely represented on the surface of different types of stem cells [60]. Subunit β1 is often associated with stem cell phenotype because, for several tissue-specific stem cells, this integrin supports the homing to stem-cell niche [61, 62], the maintenance of stemness [63], and the quantity of stem/progenitor cells in tissue [64].

There is no exception in the case of MSC. It has been shown that integrins α2β1 or α11β1 provide adequate interaction of human bone marrow MSC with type I collagen, which ensures cell survival and osteogenic differentiation by activating the protein kinase B (PKB/Akt) survival pathway [65]. Similar results were obtained in the study of integrin α5 activated signaling cascades in human bone marrow MSC during osteoblast differentiation. In this case, osteogenic differentiation of human bone marrow MSC was mediated by activation of FAK/ERK1/2 MAPKs and PI3K signaling pathways [66, 67]. Knockdown of α2 integrin in human bone marrow MSC during osteogenic differentiation on stiffer matrices was downregulated by ROCK, FAK and ERK1/2 axis [68]. Involvement of integrin α2 in human bone marrow MSC osteogenesis through activation of the p38 MAPK pathway was also demonstrated [69]. Results of another study showed that integrins in rat bone marrow MSC activate FAK-GSK3β phosphorylation, which prevents β-catenin degradation and nuclear translocation to bind to the wnt1 promoter [70]. In addition, silencing of the β1 subunit reduces both osteogenic and chondrogenic differentiation of human bone marrow MSC [71].

### Discoidin domain receptors

Discoidin domain receptors (DDRs) (DDR1 and DDR2) are collagen-binding receptors in mammals [72]. Several articles have described the structure and existing isoforms of DDRs [73–76]. DDRs are transmembrane proteins with receptor tyrosine kinase (RTK) activity [74, 76]. They are an unusual subfamily of RTKs. In comparison with typical RTKs, DDRs bind a large ligand–collagen, which stimulates autophosphorylation over several hours and forms dimers in the absence of a ligand [77]. The detection of microenvironment stability is included in DDR functions, as ectodomain shedding mediated by matrix metalloproteinases regulates the level of DDRs on the cell surface [73, 78, 79].

Additionally, DDR1 demonstrated regulation of collagen transcription by translocating to the cell It appears that DDRs regulate the directed differentiation of progenitor cells. The depletion of DDR1 expression in human adipose-derived MSC suppresses chondrogenic differentiation by decreasing the chondrogenic genes and cartilaginous matrix deposition [80]. Furthermore, DDR1 regulates the terminal differentiation of human articular chondrocytes [81]. DDR2, as a collagen receptor, plays a crucial role in regulating bone development. DDR2 knockout in limb bud chondroprogenitors inhibited chondrogenic and osteogenic differentiation [82]. Moreover, DDR2 was found as one of the potential markers of osteoblastic progenitors derived from periosteum [83]. The expression of DDR2 and integrin α11β1 was increased when human bone marrow MSC were cultured on a substrate with type 1 collagen. It was found that the expression of integrin α11β1 prevailed during chondrogenic differentiation, while the expression of DDR2 was also significantly higher during osteogenic differentiation [84].

### CD44

CD44 is primarily a receptor for hyaluronic acid (HA) but can also bind to several ligands, such as ECM components (fibronectin, laminin, osteopontin, HA), as well as some cytokines and growth factors [85]. CD44 represents a family of non-kinase transmembrane protein receptors. The structure and functions of various CD44 isoforms have previously been considered [85–87].

CD44 is a participant in multiple ECM-induced signaling pathways [85, 87] being also a well-known stem cell marker because it is represented in many stem-cell niches [88–90]. The activation of certain signaling pathways through CD44 is conditioned from the molecular weight of HA. CD44 is responsible for the migration/homing and differentiation of stem cells [85, 91–93]. The Wnt-induced/β-catenin signaling pathway is crucial for MSC commitment in osteogenic differentiation [94], and CD44 has a complex role because it is one of the gene targets and regulators of Wnt activation [85]. Moreover, CD44 is a key regulator of chondrogenic differentiation of human adipose-derived stem cells and human amniotic MSC via ERK1/2 signaling [95–97]. When the HA molecular weight is higher than CD44 human adipose-derived MSC form clusters, which stimulates chondrogenesis via ERK/SOX-9 pathway [95]. Moreover, CD44 inhibits apoptosis and enhances cell survival by ERK signaling in mouse bone marrow derived MSC and human chodrocytes [92, 98].

### Proteoglycans

Proteoglycans are ubiquitous components of the cellular microenvironment, which includes several families. They also act as membrane-bound receptors. These molecules include heparan sulfate proteoglycans (HSPGs), comprising two distinct families: syndecans (4 members) and glypicans (6 members) [99, 100]. Numerous articles give detailed descriptions of the structure and functioning of HSPGs [99, 101–106].

#### Syndecans

There is abundant evidence that syndecans activate different signaling pathways. Studies show that syndecans have no kinase activity, but other kinases can phosphorylate their intracellular domains [105]. The interaction of syndecans with different signaling pathways became obvious during shedding processes. The results of these processes showed that the representation of syndecans on the cell surface reduced, and many growth factors lost their possible binding sites on the heparan sulfate chains and showed a decreased affinity to their receptors [73].

SDC-1 knockdown in mice leads to the inhibition of canonical Wnt signaling because of deficient levels of β-catenin [107]. Temporary knockdown of SDC-1 by RNA interference in primary human MSC cultures revealed a pro-adipogenic phenotype with enhanced osteoblast maturation. These findings implicate SDC-1 as a facilitator of balance during early induction of adipoblast and osteoblast lineage differentiation [108]. Overexpression of SDC-2 in mice decreased osteoblastic and osteoclastic precursors in bone marrow, as well as Wnt/β-catenin signaling in osteoblasts [109]. SDC-3 increased the canonical Wnt signaling that controls murine osteoblast maturation in vivo [110].

#### Glypicans

Glypicans are globular glycosylphophatidylinositol (GPI) anchored proteins [99, 111]. Typically, glypicans have an N-terminal cysteine-rich domain similar to that of Frizzled receptors and mediate Wnt binding [101]. Glypicans, like syndecans, are affected by shedding. Particularly, Notum lipase cleaves glypicans and inhibits Wnt signaling [112]. GPC3 lacking mutation in mouse embryos reduced Wnt/JNK signaling [113]. GPC6 inhibited the activity of the Wnt signaling pathway in GPC6-null mice [114]. A hedgehog signaling pathway in mouse GPC3 null embryos was hyperactivated [115]). It was observed that dysregulation of glypican in human bone marrow MSC isolated from tissues of osteoarthritis patients decreased the protein level of NOTUM (extracellular negative regulator of the WNT/β-catenin signaling pathway) during osteogenic differentiation [116].

### Glycosphingolipids

Glycosphingolipids (GSLs) are amphiphilic membrane lipids of the eucaryotic plasma membrane consisting of glycan chains that are covalently linked to the sphingolipid backbone [117, 118]. GSL-associated glycans range from one and to more than 20 sugar residues, with 11 different monosaccharide types being used in vertebrates [119].

Numerous in vivo studies have reported that the composition of GSLs in the plasma membrane varies depending on the embryonic stage [117] Similar changes in GST expression can also be observed during stem cell differentiation in vitro. Thus, the GSLs composition of MSC dynamically changes according to the direction of lineage differentiation: MSC express SSEA-3 (stage-specific embryonic antigen), SSEA-4 along with GD1a and GD2 gangliosides, whereas the major GSLs of MSC-derived adipoblasts have GM3 and GD1a, MSC-derived chondrocytes have GM3 and GD3 [117, 120, 121]. A subpopulation of SSEA3( +) cells was isolated from MSC [122]. These cells, known as multilineage-differentiating stress-enduring (MUSE) cells, are endogenous and express pluripotency master genes and their capability to differentiate into cells of the three embryonic layers was established. At the same time MUSE are able to withstand stress and have an excellent ability to repair DNA damage [122, 123]. Recently, SSEA-3 was shown to act as an FGF-2 co-receptor in MUSE cells isolated from human bone marrow MSC. Their interaction activates the FGF-2 signaling cascade through PI3 kinase [122]. GM3 treatment enhanced TGF-β signaling through SMAD 2/3 during the chondrogenic differentiation of human synovial-derived MSC [124].

### Mechanosensitive ion channels

Mechanosensitive ion channels in mammals include transient receptor potential channels (TRP) and Piezo channels [125]. TRP channels comprise eight subfamilies, i.e., TRPA (ankyrin), TRPC (canonical), TRPM (melastatin), TRPML (mucolipin), TRPP (polycystin), TRPV (vanilloid), TRPN (Drosophila No mechanoreceptor potential C), and TRPY (yeast). Only the first six are found in mammals [126]. The structures of these channels have been described in detail in various works [127, 128]. Piezo channels are nonselective cationic mechanosensitive channels, which include two members, i.e., Piezo1 and Piezo2 channels [129]. Various scientific publications describe the structure and features of functioning Piezo channels [130–135].

In the various stem cell niches MSC are involved in the microenvironmental control process. TRPV4 modulates the formation of collagen fibrils by human bone marrow MSC. The inhibition of this channel in MSC disrupts aligned collagen matrix assemblies. In contrast, TRPV4 activation promotes collagen deposition [136].

Tissue-specific Piezo2 isoforms are formed as a result of alternative splicing, where each isoform may have its own specific function. The diversity of splice isoforms is performed in the neuronal tissue and cells. Only one splice isoform is expressed in non-neuronal tissue [125]. Piezo1 regulates cross-talk between osteoblasts and osteoclasts in mice. In the osteoblastic cells, the expression of type II and IX collagens is controlled by Piezo1-YAP-signaling axis. The deficiency of these collagen isoforms results in an increase in osteoclast differentiation and bone resorption in vivo [137]. Furthermore, mouse bone marrow MSC with Piezo-channel loss demonstrated the inhibition of osteoblast differentiation because of a reduction of YAP and β-catenin [138]. The effect of mechanosensitive channels on stem cell behavior is disclosed in more detail in reviews [139, 140].

Based on the studied scientific sources, we arrange a general table of known intracellular signaling pathways from ECM receptors in stem and committed progenitor cells with several examples of their realization in MSC (Table 2).

**Table 2:** Interactions of stem cell functions and signaling pathways for major ECM receptors

Receptor	α1β1	β1	α2β1	α5β1		α11β1
Signalling pathway	?	Wnt/β-catenin pathway	Protein kinase B (PKB/Akt) survival pathway; MAPK p38 signaling; ROCK, FAK, and ERK1/2 signaling axis; ?	Wnt/β-catenin signaling pathway; PI3K, FAK, and ERK1/2 signaling axis; ?		Protein kinase B (PKB/Akt) survival pathway
Stem cell function	Osteogenic differentiation on nanotopography surface	Chondrogenic differentiation and maintenance of cell phenotype; Osteogenic differentiation; Chondrogenic differentiation under fluid shear stress	Viability and osteogenic differentiation; osteogenic differentiation; osteogenic differentiation on matrices with 42.1 ± 3.2 kPa; Chondrogenic differentiation under fluid shear stress	Osteogenic differentiation on the tunable polyacrylamide hydrogels coated with fibronectin and Young’s modulus 62–68 kPa; Osteogenic differentiation; Chondrogenic differentiation under fluid shear stress		Viability and osteogenic differentiation
Stem cell type	Rat bone marrow MSC [141]	Mouse chondrocytes and rat bone marrow MSC [70]; human bone marrow MSC [71]; Rat chondrocytes [142]	Human bone marrow MSC [65, 68, 69]; Rat chondrocytes [142]	hMSC [67]; human bone marrow MSC [66]; Rat chondrocytes [142]		Human bone marrow MSC [65]
**Receptor**	**DDRs**		**CD44**	**Syndecans**		**Mechanosensing ion channels**
	**DDR1**	**DDR2**		**SDC-2**	**SDC-3**	**TRPV4**
Signalling pathway	Indian hedgehog/Gli1/Gli2/Col-X pathway	p38 MAPK kinase pathway	ERK1/2 signaling	cortactin and Survivin axis; Wnt/β-catenin signaling;	WNT signaling	TRPV4 and SOX9 axis; TRPV4/ERK/RUNX2; TRPV4 increased intracellular Ca2 + , NFATc1 nuclear translocation, and Wnt/β-catenin signaling
Stem cell function	terminal chondrocyte differentiation	osteoblast mineralization	Chondrogenic differentiation	migration; control population of osteoblastic and osteoclastic precursors in bone marrow;	osteoblast maturation	Chondrogenic differentiation; Osteogenic differentiation
Stem cell type	mouse chondrocytes [81]	rat osteoblasts [143]	human amniotic mesenchymal stem cells (hAMSC) [96]; rat and human adipose derived MSC [95]	human bone marrow MSC [93]; mice osteoblastic and osteoclastic precursors [109]	osteoblastic progenitors [110]	murine MSC [144]; Murine bone marrow MSC [145]; Rat bone marrow MSC [146]s

The symbol “?” mean unclear signaling pathway for specific process

## Potential therapeutic strategies targeting ECM-induced signaling in postnatal stem cells for regenerative medicine

Among the promising regenerative medicine strategies involving ECM receptors, two main approaches can be distinguished: 1) blocking of receptors to ECM components and their intracellular signaling pathway, and 2) activation of ECM receptors by functionalization of various surfaces and materials with specific peptides or ECM components.

It is well-known that integrins are involved in the tissue regeneration processes. The role of MSC-expressed integrins for each tissue type remains a promising area of research. Thus, following an injury, MSC are recruited from surrounding tissue to the injury site in an integrin-dependent manner [147]. Some recent studies have shown that the expression of certain integrins in MSC ensures the maintenance of the blood–brain barrier in vivo models of traumatic brain injury or ischemic stroke [148, 149]. Integrins also play a vital role in wound healing. According to the results of several preclinical studies blocking the integrins αvβ5, αvβ3, α3β1 effectively decreased myofibroblast differentiation of human dermal fibroblasts in vitro and murine lung fibroblasts in vivo in association with TGFβ signaling [150–152]. Evaluation of therapeutic success of biomaterials which functionalized with integrin-targeting peptides has been recently reviewed [147, 153]. In this context, it has been repeatedly shown that human bone marrow MSC increase the expression of osteogenic markers if cultured on materials modified with RGD-peptide (adhesion site of fibronectin) [154, 155]. Similar results were obtained for human bone marrow MSCs in osteogenic differentiation using materials functionalized with collagen-based cell adhesion motifs: GFPGER (binding integrins α1 and α2), GFPGEN (binding integrin α1 only), GFOGER (a2b1 integrin) [69, 156, 157]. Key features of integrin-binding peptides in combination with biomaterials and their strong potential as biomimetic tools for regenerative medicine are described in a recent review [158].

The role of DDRs in wound healing is still poorly understood. It is known that DDR2 regulated wound healing by activating p38 and ERK1/2 kinases and inducing matrix metalloproteinase (MMP) expression [159]. Recent studies demonstrated the crucial role of DDR2 in the regeneration of cranial bone [160]. For integrins, materials conjugated with specific collagen sequence peptides have been developed that stimulate DDR activation. For example, the GVMGFO peptide interacts with the DDR2 receptor, leading to DDR2 Y740 phosphorylation and stimulation of osteoblast differentiation [161]. A potentially novel approach in regenerative medicine is the use of extracellular vesicles or exosomes from the mouse adipose derived MSC secretome to restore ECM receptor expression. It has been shown that MSC-derived exosomes can accelerate cutaneous wound healing by suppressing miR-96-5p and restoring DDR2 expression [162].

Previously, it was shown that MSC encapsulated in HA-based hydrogels expressed more markers of cartilage tissue both in vitro and in vivo compared to control samples [163]. This was also confirmed by a study using antibody CD44 blockade, which led to the offset of this effect when MSC were cultured on HA hydrogels [164]. A recent study demonstrated the efficacy of combined therapy on angiogenesis in ischemic diseases using HA with human umbilical cord blood-derived endothelial colony-forming cells and human umbilical cord derived MSC [165].

Syndecans and glypicans regulated the normal regeneration of different tissues [166–169]. SDC-3 increased new bone formation in vivo [110]. SDC-4 is essential for regenerating damaged muscle in mouse model [170]. Exosomes derived from rat bone marrow MSC showed chondroprotective effects through the regulatory role of exosomal microRNA-9-5p (miR-9-5p) to inhibit syndecan-1 in a rat model of osteoarthritis [171]. At the same time, SDC3 deletion enhances the efficacy of murine bone marrow MSC treatment of inflammatory arthritis in vivo [172]. Among other materials alginate hydrogels containing integrin and syndecan binding peptides (cyclic RGD and AG73, respectively) were developed which exhibited higher human nucleus pulposus (NP) cell viability, biosynthetic activity, and NP-specific protein expression than alginate alone [173].

Currently, there are not many publications on the use of MSC glycosphingolipids in the field of regenerative medicine. However, some data support the idea that glycosphingolipids could be a promising target for the treatment of various diseases [174, 175].

Mechanotransduction is also involved in regenerative processes. For example, the hematoma formed after a bone fracture is highly viscoelastic, and such viscoelasticity is necessary to allow infiltration of MSCs and osteogenic stimulation of MSC in vivo [176]. Cultivation of MSC in RGD-coupled alginate hydrogels leads to the activation of TRPV4 ion channels and then nuclear translocation of RUNX2 which drives osteogenic differentiation [177].

Biofunctionalization of tissue engineering materials improves cellular interaction and tissue integration. Currently, a large number of functionalization methods of tissue engineering materials are known, ranging from single peptides and components of ECM, including enzymes responsible for its remodeling, to decellularization of tissues and whole organs, which is described in detail in the reviews [158, 178–180]. The success of the approach to obtain ECM secreted by a specific cell type, such as MSC, can be assessed based on recent studies in this area [181–183]. Furthermore, the use of ECM-derived materials for tissue repair targeting the stem cell differentiation including MSC is also a promising approach that has been confirmed by several registered clinical trials [184–187].

## Predicting the ECM receptor networks for the signaling pathway using the system biology approaches

Several approaches of network biology are widely used to study the activation of contributing factors in intracellular signaling cascades, which can subsequently find application in predictive medicine [187]. Nowadays, computational network modeling is used to determine niche-induced signaling pathways that identify stem cell outcome determinants [188]. Several network models that are used to help assess the effects of drugs on key signaling participants have been created for cancer cells [189]. The application of network biology approaches in the context of signaling pathways from the ECM led to the consideration of the integrin adhesome, which are responsible, together with integrins and integrin-associated proteins, for stabilizing cell adhesion and signal transduction [190, 191]. In addition, the “consensus adhesome” was formulated based on 60 proteins, defined by merging mass spectrometry datasets obtained from three cancer cell lines, telomerase-immortalized human foreskin fibroblasts and mouse embryonic fibroblasts attached in fibronectin-coated dishes [192].

Subsequently, with the development of omics technology and the rapid evolution of big data analysis, different approaches and specialized databases have been created by the scientific community. This is also relevant to matrix biology. One such database comprising ECM component adhesomes and interactions is MatrixDB (http://matrixdb.univ-lyon1.fr/) [193]. Another database is MatrisomeDB, which integrates experimental proteomic data on the ECM composition of normal and diseased tissue types (https://matrisomedb.org/) [194]. Recent studies reported the creation of a database (MatriNet) that will enable the study of structural changes in ECM network architectures as a function of their protein–protein interaction strengths across 20 different tumor types (www.matrinet.org) [195].

For the construction of protein–protein interactions (PPIs), many investigators are using databases that aggregate standard information from large resources (e.g., PDB, IntAct, BioGRID, HPRD). Examples of such PPI databases are STRING and Mint (http://www.string-db.org) [196, 197]; STRING was used to create molecule interactions for syndecans and Piezo channels [104, 198].

**Fig. 1:**
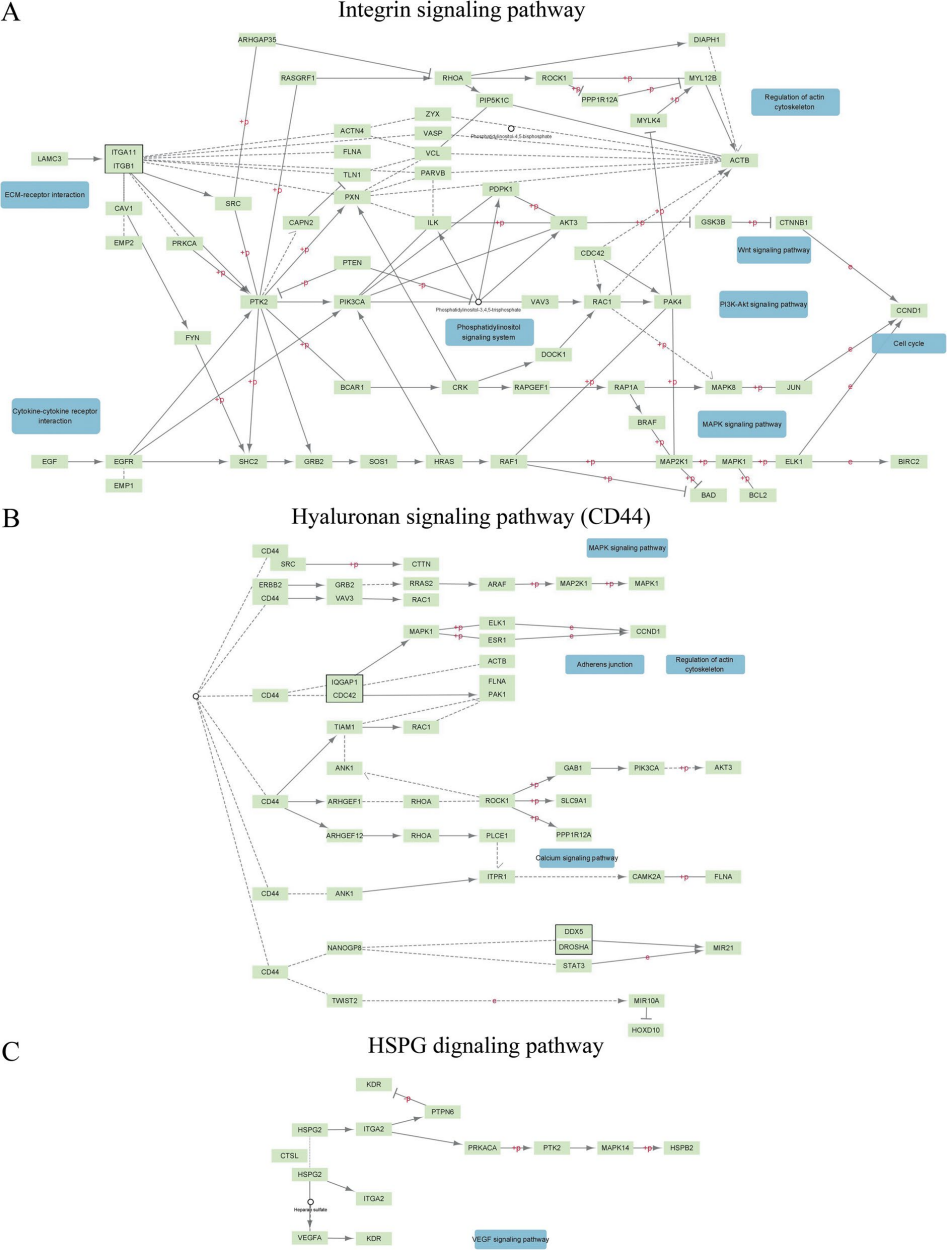
General networks of signaling pathways from ECM receptors obtained from the KEGG PATHWAY Database: **A**—integrins, **B**—CD44, and **C**—HSPG

In our review, prior to creating our PPI networks for ECM receptors, we checked preexisting networks of signaling pathways from ECM receptors in the KEGG PATHWAY Database (https://www.kegg.jp/kegg/pathway.html). We revealed the signaling pathways for integrins, CD44, and HSPG (Fig. 1).

General networks for ECM receptors included a small quantity of signaling molecules or signaling members, from receptors to growth factors, and demonstrated crosstalk between them. However, it gives rise to new difficulties in determining the function of ECM signaling pathway participants. Here, we decided to try using an approach for the definition of specific signaling pathways for individual ECM receptors.

First, we created PPI networks for integrin β1 and CD44 as one of the well-studied surface markers of stem cells and employed DDR1 as an example of a less-investigated receptor but potentially useful for application to mechanosensing. PPI networks were created using the STRING database with the following settings: network type – physical subnetworks; meaning of network edges – evidence; active interaction sources; experiments; minimum required interactions score – 0.4, 2 shell; maximum number of interactors to show – 150. In addition, Cytoscape was used for analysis and network visualization (v.3.9.1; https://cytoscape.org/) [199]. As mentioned above, the majority of data utilizing omics technology have to date been obtained from cancer cells and tissue. We verified how many signaling molecules would be excluded from our data for network construction if we used only data obtained from isolated human primary cells or human cell lines, excluding cancer cells or cell lines obtained from patients with disease, on the PPIs network for integrin β1 (Fig. 1 Supplement). As a result of this analysis, we found that only 13 of 23 members of different types of signal transduction pathways met in normal cell lines.

**Fig. 2:**
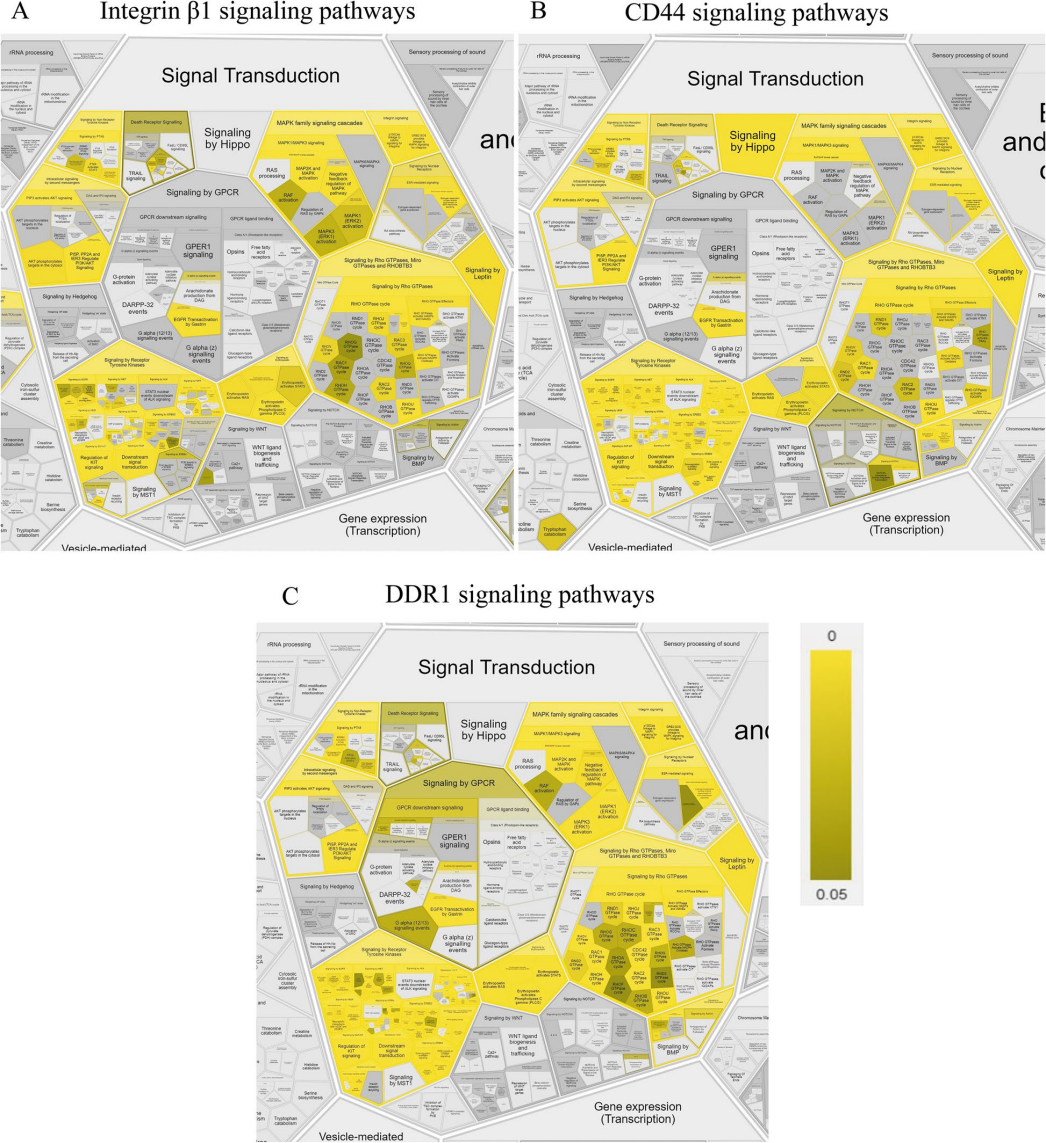
Illustration of contributing factors of the signaling pathway for integrin β1 (**A**), CD44 (**B**), and DDR1 (**C**), which generated using ReactomeFIPlugIn in Cytoscape. The obtained results are presented as part of Reactfoam according to the type of signaling pathways using a false discovery rate (FDR) scalebar (*p*-value ranging from 0–0.05)

Next, we analyzed the contributing factors of the signaling pathway for integrin β1, CD44, and DDR1 using ReactomeFIPlugIn in Cytoscape, which accessed the Reactome pathways stored in this database (https://reactome.org/) [200]. We selected the signal transduction database to analyze our networks, and the obtained results are presented as Reactfoam (Fig. 2 Supplement) as an illustration of analysis results using a false discovery rate (FDR) scalebar (p-value ranging from 0–0.05) according to not only the type of signaling pathways (Fig. 2) but also cellular function (Fig. 2_Supplement).

**Fig. 3:**
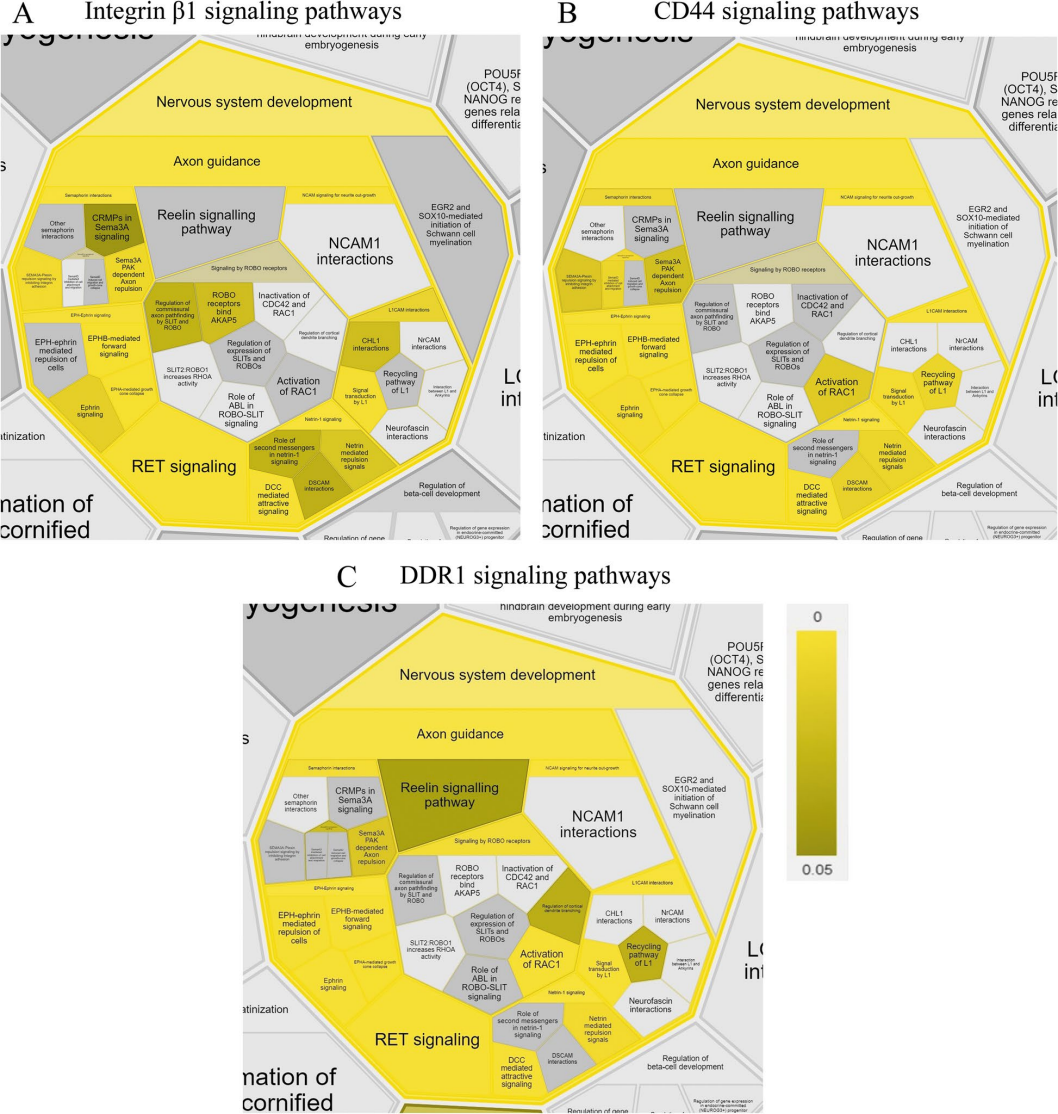
Illustration of contributing factors of the signaling pathway for integrin β1 (**A**), CD44 (**B**), and DDR1 (**C**), which generated using ReactomeFIPlugIn in Cytoscape. The obtained results are presented as part of Reactfoam according to the cellular function using a false discovery rate (FDR) scalebar (*p*-value ranging from 0–0.05)

We reveal that the signaling pathway members of integrin β1, CD44, and DDR1 are vital for nervous system development (Fig. 3).

**Fig. 4:**
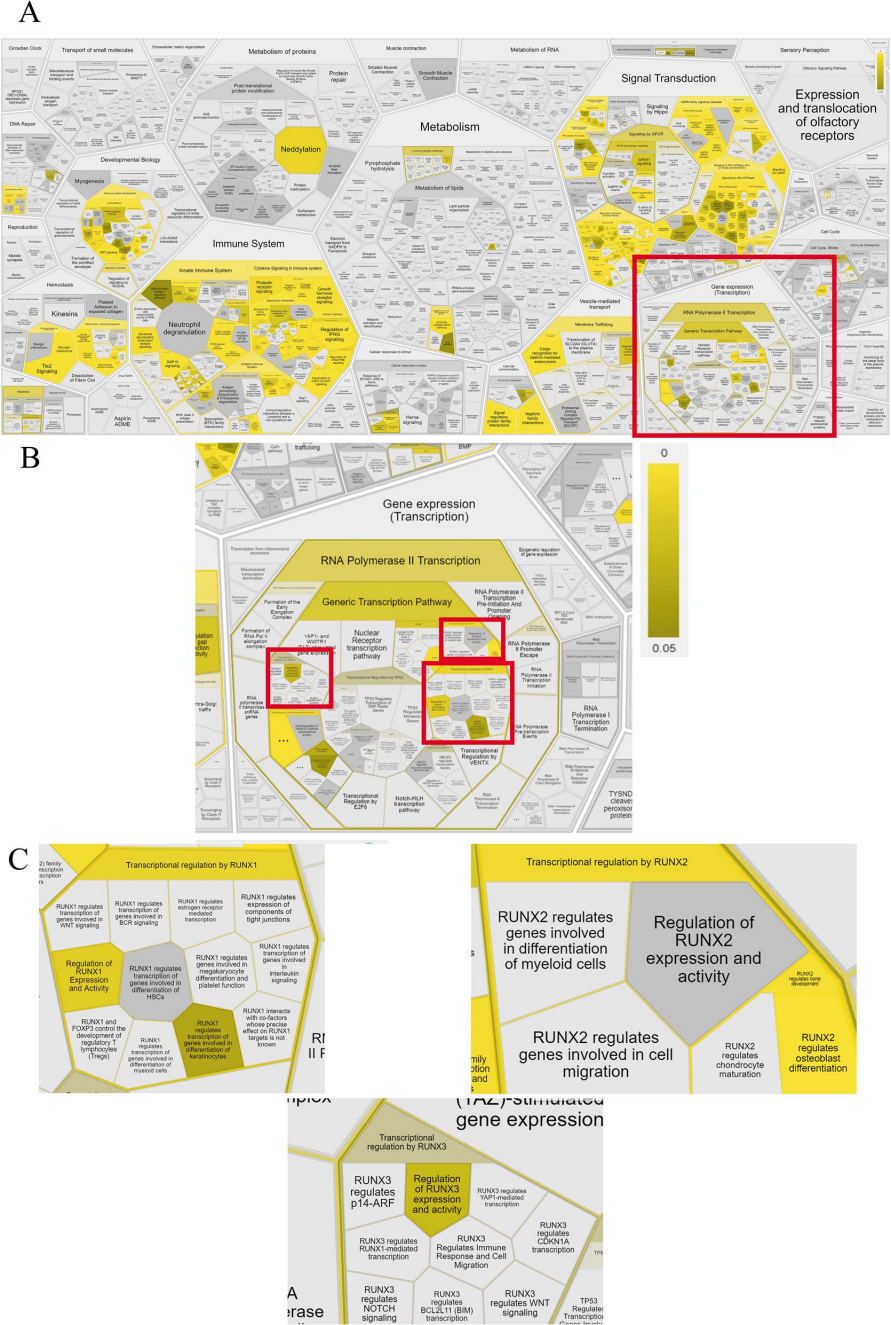
Illustration of contributing factors of the signaling pathway for DDR1 in human adipose-derived MSC (**A**), (**B**), (**C**), which generated using ReactomeFIPlugIn in Cytoscape (**B** and **C**—enlarged parts of the image **A**). The obtained results are presented as part of Reactfoam according to the cellular function using a false discovery rate (FDR) scalebar (*p*-value ranging from 0–0.05)

In addition, we build a prediction network of PPIs for DDR1 pathway members in human adipose-derived MSC. For this step, we normalized the data of PPIs using RNAseq data describing the transcriptome of human adipose-derived MSC obtained in our laboratory (Fig. 4).

## Conclusions

ECM-induced signaling pathways within the stem cells may define the functionality and homeostasis of the whole tissue. Trying to understand the complicated molecular interactions between ECM and stem and progenitor cells, scientists are developing new approaches based on a general view obtained from big data analysis. The application of system biology methods for this purpose has been an observable current trend in matrix biology [201].

In this review, we discussed the results of recent studies covering the participation of cellular receptors to ECM components in the maintenance of postnatal tissue homeostasis as well as tissue regeneration after various types of damage through the regulation of MSC functions. We also focused on summarizing the data of the currently known ECM-induced signaling cascades in human stem and progenitor cells. However, literature analysis revealed that very few and only the most common participants of these signal pathways are investigating; moreover, they are involved in multiple cellular processes which makes them not suitable as a potential therapeutic target to modulate stem cell functioning for the purposes of regenerative medicine. Therefore, to detect more specific ECM-induced signaling pathways in stem and progenitor cells as well as to search for novel targets for fine-tuning regeneration processes, we suggest using the approaches of systems biology.

**Fig. 5:**
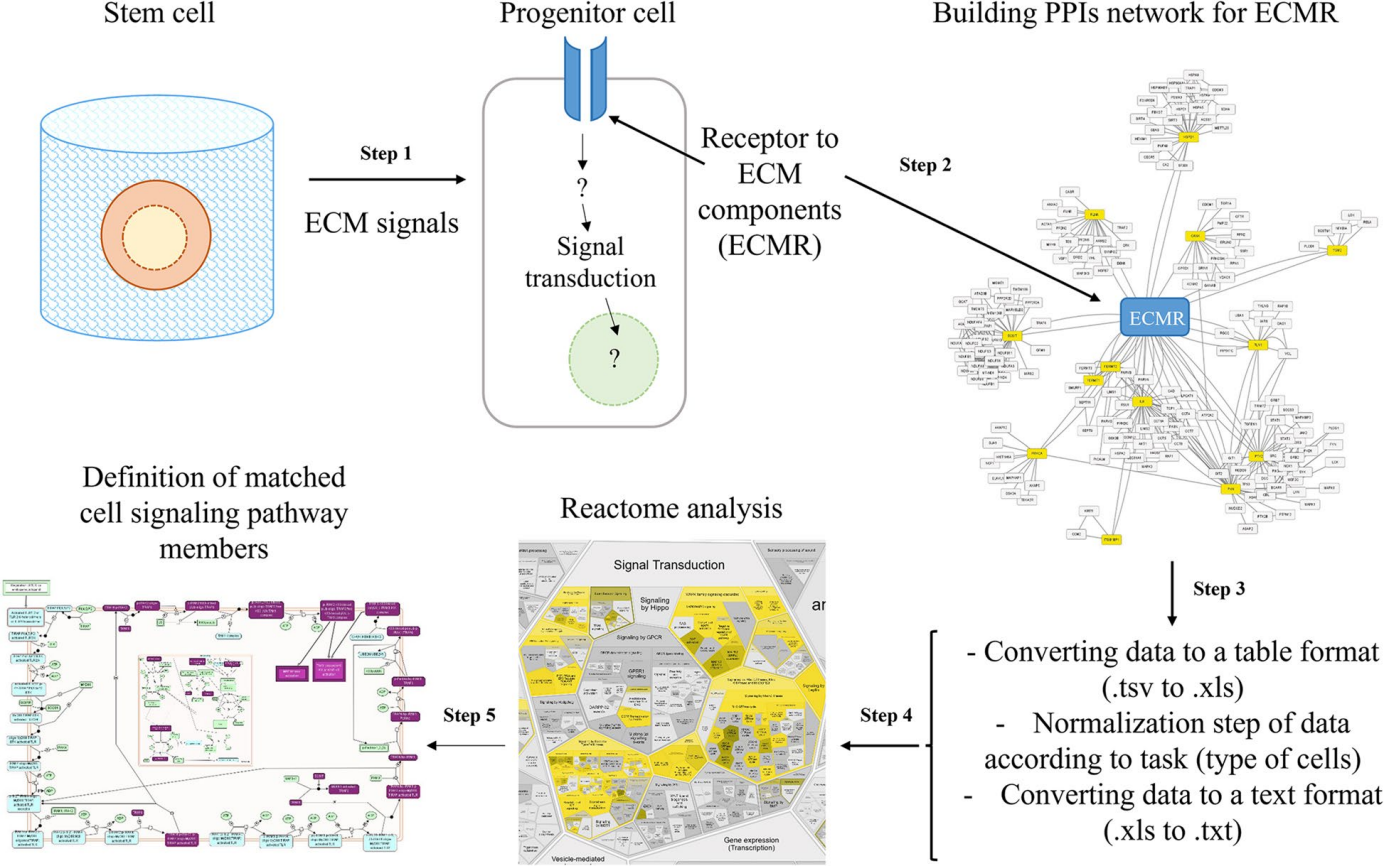
Summary illustration of review

Utilizing the established changes in the stem cell profile of ECM receptors (Step 1), it is possible to build predictive networks of PPIs (Step 2). Such networks can be analyzed based on the well-known and available databases described in this review. After receiving the list of participants in the signaling cascade, a normalization step can be added to the data of the proteome or transcriptome of cells of interest to researchers (Step 3). Then, the created network can be compared with a database of known signal pathways to select specific signaling pathways and processes accumulating the most of participants from the previously predicted network of PPIs (Step 4 and Step 5). The resulting theoretical model could be useful in designing the further experimental research exploring the ECM-mediated regulation of stem and progenitor cells (Fig. 5).

This review is the first to detail the major types of ECM receptors in postnatal stem cells, using MSC as an example, and to evaluate the involvement of ECM-induced signaling cascades in the process of MSC differentiation. In addition, a detailed algorithm of action using state-of-the-art methods in the field of systems biology is proposed to explore the variability of ECM signaling pathways in different cells. It may help other researchers in the field to discover new targets for tissue and organ regeneration with the ability to fine-tune cellular mechanisms rather than inhibiting members of cascades that are responsible for multiple processes and could potentially lead to pathology or cell death (e.g., such as Src, Erk, Akt).

Shortly, we suppose that the suggested approach of generating the predictive networks of PPI could serve as a useful tool which is complementary or even partially replacing the omics experimental work. Combining the known data about ECM receptor on the specific target cells and desirable functional processes one could reveal novel expected or unexpected signal transduction pathways induced by ECM. Few examples are demonstrated in the review. Indeed, these results have limited value until experimental validation. However, the suggested algorithm could intensify the search of ECM-induced signaling pathways in stem and progenitor cells and shorten the time to the potential breakthroughs in the field.

Thus, following the current trends in the field of matrix biology, it could allow to identify new promising directions in the study of stem and committed cell behavior within their matrix microenvironment. Importantly, these approaches expand the number of tools in regenerative medicine using to search for the mechanisms regulating tissue and organ renewal and repair.

### Supplementary Information

**Additional file 1: Fig. 1_Supplement. **Predicting the integrin β1 PPIs networks for signal transduction molecules before (A) and after (B) excluded data obtained from cancer cells.

**Additional file 2: Fig. 2_Supplement. **Illustration of contributing factors of the signaling pathway for integrin β1 (A), CD44 (B), and DDR1 (C), which generated using ReactomeFIPlugIn in Cytoscape. The obtained results are presented as Reactfoam using a false discovery rate (FDR) scalebar (p-value ranging from 0–0.05).

**Additional file 3: Fig. 3_Supplement. **Predicting the DDR1 signaling pathway members participate in semaphorin interactions (A), and regulate the activity of RUNX1, RUNX2, and RUNX3 transcription factors in the case of osteogenic differentiation (B).

## Data Availability

All data generated or analyzed during this study are included in this published article [and its supplementary information files].

## Abbreviations

CD44: Cluster of differentiation 44
DDR1: Discoidin domain receptor tyrosine kinase 1
DDR2: Discoidin domain receptor tyrosine kinase 2
ECM: Extracellular matrix
FAK: Focal adhesion kinase
GPI: Glycosylphophatidylinositol
GSLs: Glycosphingolipids
HA: Hyaluronic acid
HSPGs: Heparan sulfate proteoglycans
IAC: Integrin adhesion complex
ILK: Integrin-linked kinase
MMP: Matrix metalloproteinases
MSC: Mesenchymal stromal cells
MUSE: multilineage-differentiating stress-enduring cells
PLC: phospholipase C
PPIs: protein–protein interactions
RNA: ribonucleic acid
RTK: eceptor tyrosine kinase
TRP: Transient receptor potential channels
