# Supplementary figures and images for: Extracellular matrix-induced signaling pathways in mesenchymal stem/stromal cells
Source: Cell Commun Signal. 2023 Sep 19;21:244. doi: 10.1186/s12964-023-01252-8 (PMC10507829; doi:10.1186/s12964-023-01252-8)

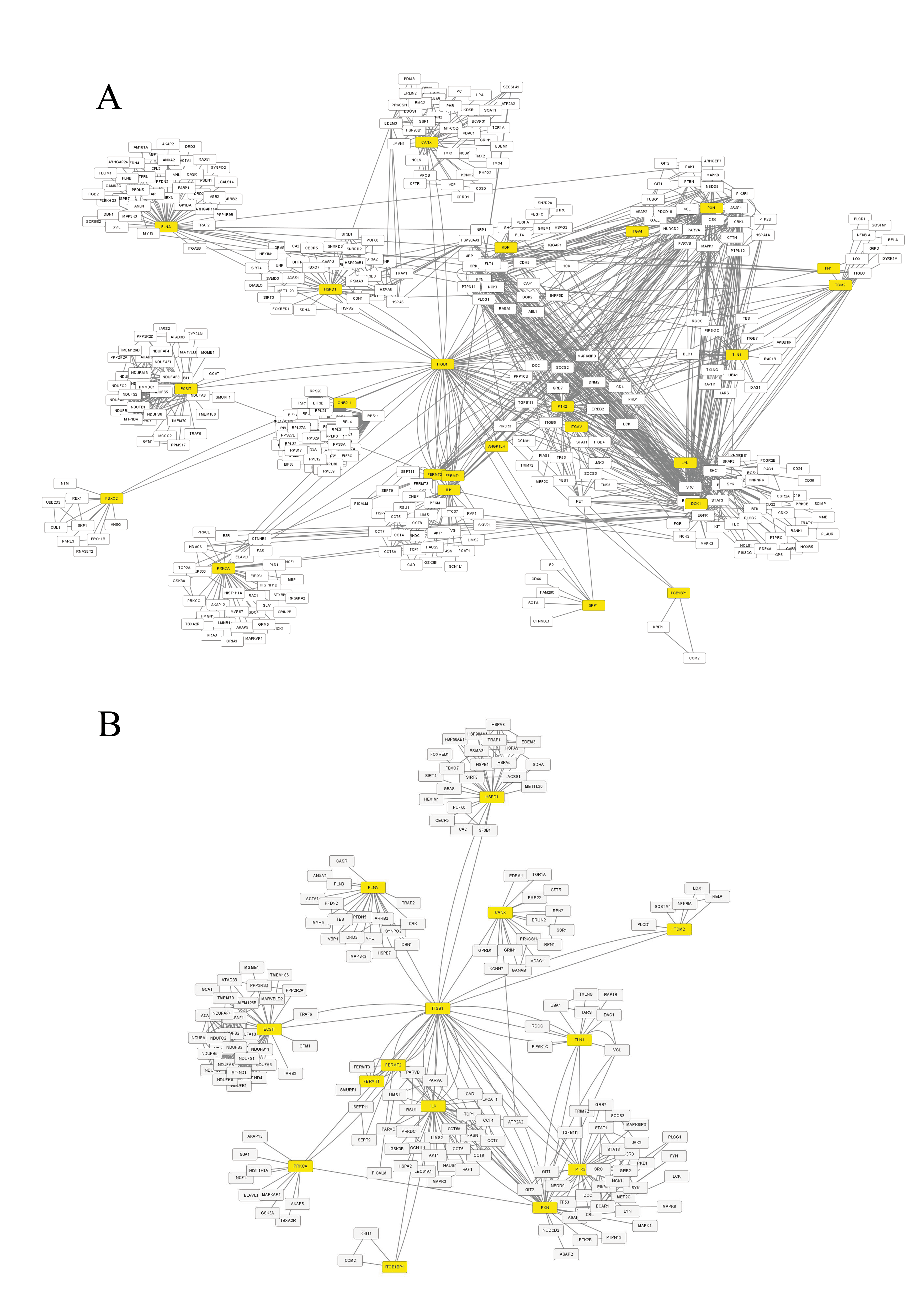

Supplement: Supplementary file 2 — Additional file 1: Fig. 1_Supplement. Predicting the integrin β1 PPIs networks for signal transduction molecules before (A) and after (B) excluded data obtained from cancer cells. [file 12964_2023_1252_MOESM1_ESM.tif]

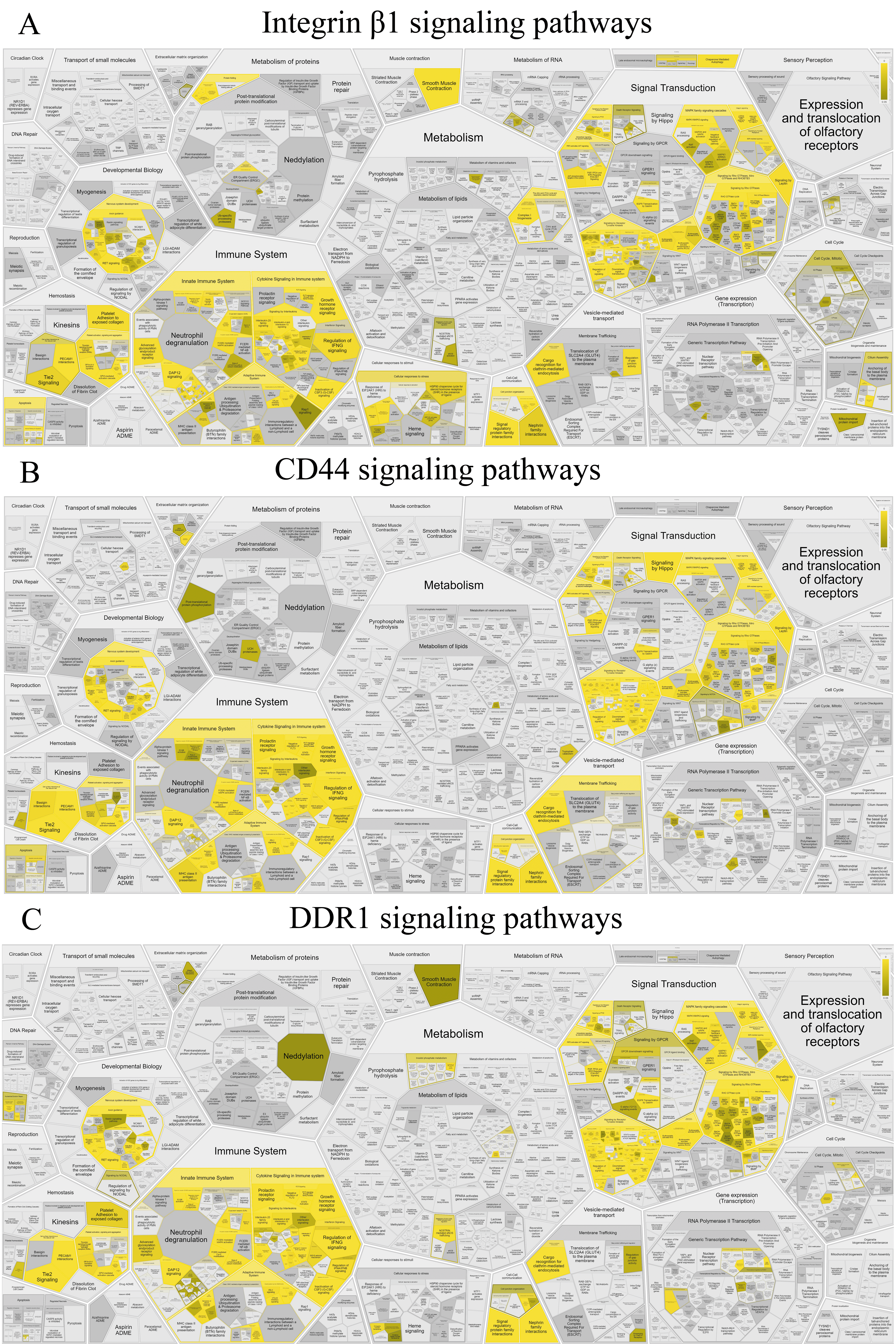

Supplement: Supplementary file 3 — Additional file 2: Fig. 2_Supplement. Illustration of contributing factors of the signaling pathway for integrin β1 (A), CD44 (B), and DDR1 (C), which generated using ReactomeFIPlugIn in Cytoscape. The obtained results are presented as Reactfoam using a false discovery rate (FDR) scalebar (p-value ranging from 0–0.05). [file 12964_2023_1252_MOESM2_ESM.tif]

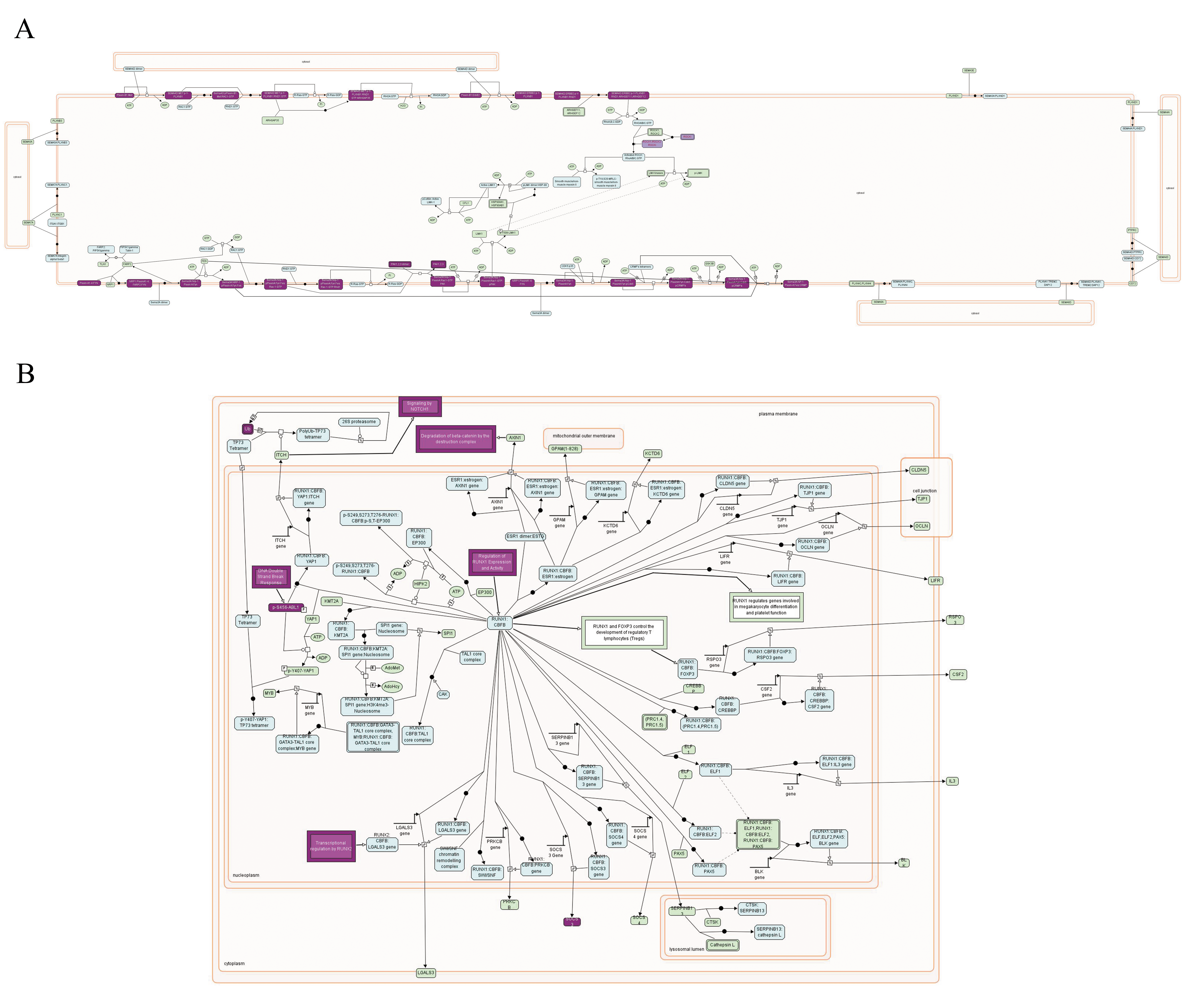

Supplement: Supplementary file 4 — Additional file 3: Fig. 3_Supplement. Predicting the DDR1 signaling pathway members participate in semaphorin interactions (A), and regulate the activity of RUNX1, RUNX2, and RUNX3 transcription factors in the case of osteogenic differentiation (B). [file 12964_2023_1252_MOESM3_ESM.tif]
